# A multi-centre phase IIa clinical study of predictive testing for preeclampsia: improved pregnancy outcomes via early detection (IMPROvED)

**DOI:** 10.1186/1471-2393-13-226

**Published:** 2013-12-07

**Authors:** Kate Navaratnam, Zarko Alfirevic, Philip N Baker, Christian Gluud, Berthold Grüttner, Karolina Kublickiene, Gerda Zeeman, Louise C Kenny

**Affiliations:** 1 Centre for Womens Health Research, University of Liverpool, First Floor, Liverpool Women's Hospital, Crown Street, Liverpool L8 7SS, UK; 2 Keele University School of Medicine, Stoke-on-Trent, UK; 3 Copenhagen Trial Unit, Copenhagen, Denmark; 4 The University of Cologne, Cologne, Germany; 5 Karolinska Institute, Stockholm, Sweden; 6 Erasmus University, Rotterdam, Netherlands; 7 University College Cork, Cork, Ireland

**Keywords:** Pre-eclampsia, Screening, Proteomics, Metabolomics, Preterm birth, Small for gestational age

## Abstract

**Background:**

5% of first time pregnancies are complicated by pre-eclampsia, the leading cause of maternal death in Europe. No clinically useful screening test exists; consequentially clinicians are unable to offer targeted surveillance or preventative strategies. IMPROvED Consortium members have pioneered a personalised medicine approach to identifying blood-borne biomarkers through recent technological advancements, involving mapping of the blood metabolome and proteome. The key objective is to develop a sensitive, specific, high-throughput and economically viable early pregnancy screening test for pre-eclampsia.

**Methods/Design:**

We report the design of a multicentre, phase IIa clinical study aiming to recruit 5000 low risk primiparous women to assess and refine innovative prototype tests based on emerging metabolomic and proteomic technologies. Participation involves maternal phlebotomy at 15 and 20 weeks’ gestation, with optional testing and biobanking at 11 and 34 weeks. Blood samples will be analysed using two innovative, proprietary prototype platforms; one metabolomic based and one proteomic based, both of which outperform current biomarker based screening tests at comparable gestations. Analytical and clinical data will be collated and analysed via the Copenhagen Trials Unit.

**Discussion:**

The IMPROvED study is expected to refine proteomic and metabolomic panels, combined with clinical parameters, and evaluate clinical applicability as an early pregnancy predictive test for pre-eclampsia. If ‘at risk’ patients can be identified, this will allow stratified care with personalised fetal and maternal surveillance, early diagnosis, timely intervention, and significant health economic savings. The IMPROvED biobank will be accessible to the European scientific community for high quality research into the cause and prevention of adverse pregnancy outcome.

**Trial registration:**

Trial registration number NCT01891240

The IMPROvED project is funded by the seventh framework programme for Research and Technological development of the EU. http://www.fp7-improved.eu/

## Background

An estimated 50 million babies are born to first time mothers worldwide every year, with 2.4 million in the 27 European Union countries [1]. Almost 1 in 20 of these pregnancies are complicated by pre-eclampsia, a disease of late pregnancy, characterized by the concomitant occurrence of hypertension and proteinuria.

The condition is associated globally with 70,000–80,000 maternal and over 500,000 infant deaths annually. For the mother it can lead to acute problems in the liver, kidneys, brain and the clotting system. Pre-eclampsia is the most important cause of maternal death in Europe, accounting for 17–24% of all maternal deaths [2]. Additionally, epidemiological studies have demonstrated that pre-eclampsia is associated with an increased risk of cardiovascular and metabolic diseases later in the mother’s life [3,4]. A quarter of the babies born to mothers with pre-eclampsia are growth restricted and a third are premature; pre-eclampsia accounts for approximately 20% of neonatal intensive care unit costs. The child may have problems with neurocognitive development that can result in mild learning difficulties through to severe disabilities. Being born growth restricted also predisposes the child to cardiovascular disease as an adult [5].

Every year, an estimated €31 billion is spent in the developed world on direct healthcare costs to provide antenatal care for nulliparous women and treatment for pre-eclampsia; of this, an estimated €9 billion is spent in Europe [6]. The healthcare costs of one case of pre-eclampsia are estimated to exceed €15,000 (all maternal and neonatal hospital costs, not accounting for longer term implications for the baby) [6]. An effective screening test would facilitate stratification and targeting of limited resources [6]. Preliminary analyses suggest that an effective test which halves antenatal visits, followed by the administration of aspirin, for screen positive women, (which reduces the incidence of disease by 20–25% [7]) would be of significant economic benefit if the unit cost of a screening test is €400–€800.

Pre-eclampsia is a heterogeneous condition with respect to the onset and severity of the clinical manifestations, this has hampered the development of screening strategies and the development and assessment of potential preventive interventions.

Circulating factors predate the clinical signs; in pre-eclampsia there are demonstrable biologically active circulating factors that are apparent well before the clinical presentation of the disease [8]. Nevertheless, there are currently no early pregnancy predictive tests for pre-eclampsia. Numerous candidate biomarkers (>200 studies so far) have been proposed for prediction of disease, including placental hormones, angiogenic factors, and lipids [9]. However, none (nor any combination) has emerged with the adequate specificity and sensitivity to be of clinical use. Indeed the World Health Organization’s (WHO) systematic review assessed the usefulness of clinical, biophysical, and biochemical tests in the prediction of pre-eclampsia and concluded that there is no cost effective or reliable screening test for pre-eclampsia [10]. Without such a screening test, clinicians are unable to offer either targeted surveillance or potential preventative therapies to those at greatest risk.

The *IMPROvED* consortium is a new and distinctive partnership of four small and medium enterprises (SMEs) and eight academic institutions, with complementary and world leading expertise. We are a diverse group of obstetric academics, laboratory and social scientists, entrepreneurs, regulators, practitioners, clinicians, biostatisticians and health economists from across Europe and beyond, and we will be supported by end-user patient support groups. All *IMPROvED* partners in this consortium are motivated by the current absence of a clinically useful screening test for pre-eclampsia. Through a multi-centre hospital-based study, representative of different healthcare models, we will establish a high calibre pregnancy bio-bank for European pregnancy researchers. We will then utilise a dual strategy of distinct but complementary cutting edge platforms to measure novel metabolomic biomarkers (MetTest) and proteomic biomarkers (ProTest) which we have previously identified as predictive of disease [11,12]. The development of such a personalised medicine approach, that offers first time mothers accurate risk assessment for pre-eclampsia, will radically impact the provision of antenatal care, both in Europe and the rest of the world, and will reduce the clinical complications of the leading cause of maternal death in Europe.

## Methods/Design

IMPROvED is a multicentre, European phase IIa clinical study, with clinical centres in Ireland, U.K, Germany, Sweden and the Netherlands. During the 2 year study period 5000 low risk, nulliparous women will be recruited between 9 + 0–16 + 6 weeks’ gestation (See details Table 1). The primary outcome is pre-eclampsia. Preeclampsia is defined as gestational hypertension (systolic BP ≥ 140 mmHg and/or diastolic BP ≥90 mmHg (Korotkoff V) on at least 2 occasions 4 h apart after 20 weeks’ gestation, but before the onset of labour or postpartum systolic BP ≥ 140 mmHg and/or diastolic BP ≥90 mmHg on at least 2 occasions 4 h apart with proteinuria (≥ 300 mg/24 h or spot urine protein:creatinine ratio ≥ 30 mg/mmol creatinine, or urine dipstick protein > = ++) (Table 2). To maximise the utility of the IMPROvED biobank for the scientific community, other primary outcomes include spontaneous pre-term birth <37 + 0 weeks, and small for gestational age babies <10^th^ customised centile (See Table 2 for exclusion criteria).

**Table 1:** Recruitment targets by centre

**Centre**	**Recruits**
Cork	1000
Keele	1000
Liverpool	500
Stockholm	750
Rotterdam	1000
Cologne	750

**Table 2:** Exclusion criteria

•	Unsure of LMP and unwilling to have USS at ≤ 20 weeks
•	≥ 3 miscarriages
•	≥3 terminations
•	Known or suspected major fetal anomaly/abnormal karyotype
•	Essential hypertension treated pre-pregnancy
•	Moderate-severe hypertension at booking (BP >160/100 mmHg)
•	Diabetes
•	Renal disease
•	Systemic Lupus Erythematosus
•	Anti-phospholipid syndrome
•	Sickle cell disease
•	HIV positive
•	Major uterine anomaly
•	Cervical suture in situ
•	Knife cone biopsy
•	Long term steroids
•	Treatment with low-dose aspirin
•	Treatment with heparin/low molecular weight heparin

### Recruitment

**Figure 1:**
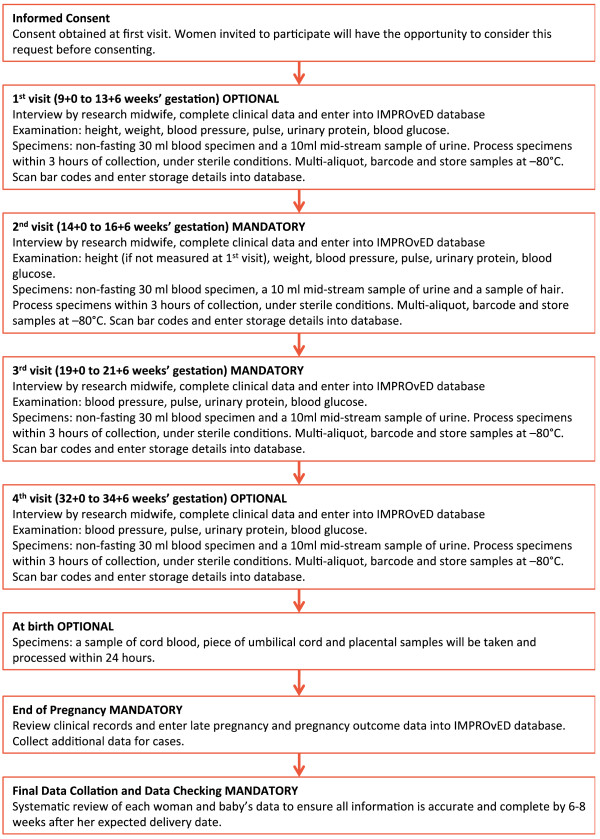
Flowchart of IMPROvED visits and pregnancy outcome data.

Logistics for recruitment will be adapted to suit individual centres participating in the study. Women will be referred through a number of routes including referral by their midwife, obstetrician or general practitioner and self-referral following exposure to the study through friends, posters, advertisements, website and news stories. Maternity caregivers in each centre will be encouraged to provide information about the study to eligible women in early pregnancy. Attempts will be made to recruit women from all socioeconomic and ethnic groups in the participating centres. Maternal age and ethnicity will be recorded on women who are approached to participate but decline, and data compared with those who consent to participate. All patients recruited must consent to sampling at the second (15 week) and third (20 week) time-points. The first (11 week) and fourth (34 week) time-points are desirable but not mandatory. Blood specimens will be collected at all sites. At certain sites urine specimens, hair from women, DNA from participants, partners and DNA samples from the baby at birth will also be collected (Figure 1).

### First sampling (optional)

At the first visit (11 + 0 to 13 + 6 weeks’ gestation), eligibility will be confirmed, informed consent signed and these data entered into the database. Maternal measurements will be performed and entered in the database. Blood specimens will be collected and information about the specimens will be entered into the database.

### Second and third samplings (essential)

At the 15-week visit (14 + 0 to 16 + 6 weeks’), the woman will be interviewed and information entered directly into the database on demographics, current pregnancy details and smoking and alcohol habits. Maternal measurements (See details Figure 1) will be performed and entered in the database. Blood specimens will also be collected and all data will be entered directly into the database.

At the 20-week visit (19 + 0 to 21 + 6 weeks’ gestation) blood specimens will be collected and maternal measurements (See details Figure 1) will be performed. All data will be entered directly into the database. Participants will be instructed to contact the research midwife if she delivers before the final visit or if she develops one of the pregnancy endpoints.

### Fourth sampling (optional)

At the final visit (32 + 0 to 34 + 6 weeks’ gestation) blood specimens will be collected and maternal measurements (See details Figure 1) will be performed. All data will be entered directly into the database. Participants will be instructed to contact the research midwife when she delivers or if she develops one of the pregnancy endpoints.

### Partner’s participation

At certain sites, participants’ partners will also be recruited. At any of the visits, or by extra appointment, partners will give one blood sample for DNA analysis.

### At birth

At certain sites, blood from the umbilical cord, a sample of the cord itself and placental samples will be taken shortly after delivery.

### Pregnancy outcome

Where possible, a research midwife will see each participant within 72 hours following delivery. Information about pregnancy events since the final visit but before the birth will be obtained. Information will also be obtained about the delivery, the baby and maternal and infant outcome in the postnatal period. If seen within 72 hours, the baby will be measured or measurements will be obtained from the medical records. The information will be confirmed by review of her medical records and entered onto the database.

Participant status at the time of delivery will be recorded in the final pregnancy outcome Case Report Form (CRF) as either continuing in the study, a fetal death, a termination after 20 weeks’ gestation or withdrawn/lost to follow up from the study. Information about pregnancy complications will also be recorded. A decision will be made as to whether each woman develops one or more of the primary endpoints. All participants who develop pre-eclampsia, deliver a SGA baby or experience spontaneous pre-term birth will have detailed clinical, laboratory and outcome data collected. Each woman and baby’s data will be systematically reviewed 6–8 weeks following her expected date of delivery to ensure accuracy.

### Sample collection and biobank

Serum and plasma specimens will be split into 0.25 ml aliquots, labelled with a unique barcode. Each barcode, along with storage details, will be entered into the database. Aliquots of different types of specimens will be colour coded and the aliquots will be stored in the -80°C freezers within 3 hours of collection. The freezers will be equipped with remote alarm systems, monitored 24/7 and study personnel responsive to any alarm. Aliquots from each recruitment centre will be transferred (at -80°C) to Metabolomics Diagnostics (Cork, Ireland) and Pronota (Ghent, Belgium) for determination of metabolomics and proteomic biomarkers and thus assessment of the performance of MetTest and ProTest [11,12]. The remaining aliquots will be transferred to the IMPROvED biobank, housed at the University of Cork, and will be available to the European scientific community for high quality research into the cause and prevention of adverse pregnancy outcome.

### Database

The database will have a biobank facility with separate forms for each type of participant specimen collected at each visit. The aliquot barcodes and biobank database will ensure that the storage position and usage of all aliquots will be able to be tracked.

The data system will be built to the same security and confidentiality standards as those of hospital electronic patient records. Innovative features of the informatics platform are that demographic, clinical, biochemical, genomic and diagnostic data can all be stored within the same architecture, and can be co-visualized for comparisons. The novel approach of personalised medicine will be applied by following a path for the individual in the biobank and clinical data through the aggregation of the information in the risk assessment algorithms, leading back to the individualised risk assessment of that particular patient. All data are stored in a secure manner within a remotely accessible electronic database.

Furthermore, the informatics platform will provide a database for the study biobank to enable rapid sample entry (by barcode) and sample retrieval. This system also allows a unique subject identifier to be linked with the sample identifiers within the biobank to allow rapid selection and to support secondary studies. A system to enable users to perform ad-hoc queries on collected data, select specimens based on ad-hoc set of clinical attributes of the subjects and their values as well as specimen features will be developed.

### Ethics

The Ethics Advisory Board (EAB) for the IMPROvED study will be chaired by Dr Deirdre Madden at University College Cork. Other members include Professor Lesley McCowan at the University of Auckland, New Zealand, Professor Robert Shaw at the University of Nottingham, UK and Xavier Carne at the University of Barcelona, Spain. The EAB will liaise closely with the Copenhagen Trial Unit and the Study Coordinator to ensure that the study is performed to the highest ethical standard.

Informed written consent will be obtained from all women participating in the study. All participating centres have obtained approval for the study from their respective ethic committees.

### Statistical analysis

We will independently examine the predictive power of ProTest and MetTest. We will mine the proteomic and metabolomic data for algorithms which have the potential to generate greater sensitivity and specificity. Any such algorithms will be incorporated into future studies.

We will also evaluate the combinatorial power of proteomics and metabolomics based tests (augmented by clinical data), to enhance the prognostic specificity and sensitivity. One of the models we will specifically explore is a sequential model of screening for pre-eclampsia, with MetTest at 15 weeks gestation followed by ProTest at 20 weeks. Such a sequential screening model is akin to that employed for Downs’ Syndrome; first trimester screening is followed by screening at 15–18 weeks. Statistical significance will be reported with both P values and confidence intervals at 95%.

### Power calculations

Power calculations have been considered extensively. Given the complexity of the study, there is no single simple solution. For the purpose of sample size estimation of the overall study, we used a binary outcome and associated measures of sensitivity and likelihood ratio as determinants of the value of these tests. Although the predictive algorithms will produce a continuous risk score, the use of a categorical outcome fits with the final binary decision process (to treat or not to treat) based on the risk score.

Based on the lowest estimated prevalence of pre-eclampsia of 3%, a test sensitivity of 93% and a specificity of 97%, then we need to recruit 4800 women to be 90% certain that the true specificity of the patient population is no less than 95%. Thus, allowing for patient dropout, a study population of 5,000 women should be sufficiently powered.

## Discussion

A considerable body of evidence demonstrates that many late pregnancy complications have their foundations in early pregnancy, and much work in pre-eclampsia has focussed on this premise [13]. However, some studies of tests for placental dysfunction indicate a temporal relationship between biochemical and ultrasonic measurements, and evolution of pregnancy complications [14]. In the IMPROvED study, obtaining samples across all trimesters will facilitate assessment of gestational age dependence for risk-assessment. Tests that can predict preeclampsia across gestations, including near term, may be more applicable to different models of antenatal care, including lower income settings [14]. Additionally, contributing biobank samples from all trimesters will provide a valuable resource for research into adverse pregnancy outcomes.

A viable screening programme requires accurate identification of women at high risk early in the disease process, and effective interventions to modify risk, and improve outcomes [14]. These fundamental components are contentious in pre-eclampsia. A sufficiently discriminatory test, has yet to be detailed. Although aspirin reduces the risk of pre-eclampsia in high risk women by 20–25%, there is no highly active disease modifying therapy.

**Figure 2:**
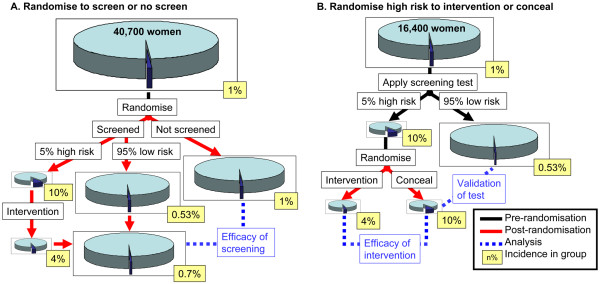
**The effect of study design on sample size calculations and conclusions that can be drawn from screening studies. (A)** Women are randomised to having or not having the screening test performed. **(B)** Women have the screening test performed and those who screen as high risk are randomised to having an intervention or having the result concealed.

Smith argues that failure to develop more effective screening methods is partly due to limitations in research methodology [14]. If cost-effectiveness of screening tests is to be evaluated in randomised trials, two study designs should be considered i.e. randomisation prior to, or after the application of a screening test (Figure 2). These two designs address very different research questions. In the context of pre-eclampsia screening, randomisation confined to screen positive women, to intervention (aspirin) vs. no intervention (placebo) allows adequate effectiveness assessment of intervention (aspirin), but not of the whole screen and treat programme. The only way to adequately assess the impact of the whole pre-eclampsia screening programme would be to compare it with the randomly allocated unscreened population for full cost-effectiveness analysis. The impact of screening tests developed in the context of the IMPROvED study will be suitable for assessment by either study design.

In summary, we describe the pragmatic design for a European multicentre phase IIa clinical study to assess the clinical applicability of predictive testing for pre-eclampsia in low risk women, and the establishment of a European biobank. The design will allow assessment of the predictive performance of the proteomic and metabolomic tests throughout pregnancy. Blood samples obtained in all trimesters will maximise the usefulness of the IMPROvED biobank for European pregnancy research.

## Abbreviations

IMPROvED: Improved pregnancy outcomes via early detection; WHO: World Health Organisation; ProTest: Proteomic test; MetTest: Metabolomic test; OR: Odds ratio; PLGF: Placental growth factor; SFlt: Soluble fms-like tyrosine kinase.

## Competing interests

LK and PB have minority shareholdings in Metabolomic Diagnostics. LK and PB are both in receipt of commercial/non-commercial monies to develop screening tests for other pregnancy complications. Otherwise, the authors declare that they have no competing interests.

## Authors’ contributions

LK conceived and designed the study with PB. LK and PB produced the detailed protocol, with input from all authors. KN drafted the manuscript with assistance from ZA and LK. All authors read and approved the final manuscript.

## Pre-publication history

The pre-publication history for this paper can be accessed here:

http://www.biomedcentral.com/1471-2393/13/226/prepub
